# Replication and transcription on a collision course: eukaryotic regulation mechanisms and implications for DNA stability

**DOI:** 10.3389/fgene.2015.00166

**Published:** 2015-04-28

**Authors:** Alessandra Brambati, Arianna Colosio, Luca Zardoni, Lorenzo Galanti, Giordano Liberi

**Affiliations:** ^1^Istituto di Genetica Molecolare del Consiglio Nazionale delle RicerchePavia, Italy; ^2^The FIRC Institute of Molecular Oncology FoundationMilan, Italy

**Keywords:** replication–transcription conflicts, R-loops, replication stress, cancer, neurodegeneration, genetic instability, epigenetic instability

## Abstract

DNA replication and transcription are vital cellular processes during which the genetic information is copied into complementary DNA and RNA molecules. Highly complex machineries required for DNA and RNA synthesis compete for the same DNA template, therefore being on a collision course. Unscheduled replication–transcription clashes alter the gene transcription program and generate replication stress, reducing fork speed. Molecular pathways and mechanisms that minimize the conflict between replication and transcription have been extensively characterized in prokaryotic cells and recently identified also in eukaryotes. A pathological outcome of replication–transcription collisions is the formation of stable RNA:DNA hybrids in molecular structures called R-loops. Growing evidence suggests that R-loop accumulation promotes both genetic and epigenetic instability, thus severely affecting genome functionality. In the present review, we summarize the current knowledge related to replication and transcription conflicts in eukaryotes, their consequences on genome stability and the pathways involved in their resolution. These findings are relevant to clarify the molecular basis of cancer and neurodegenerative diseases.

## Introduction

DNA replication and transcription are vital processes in all living organisms during which specialized polymerases copy the genetic information into complementary DNA and RNA molecules. Both processes must be completed with high fidelity to preserve genetic information and cell functionality. The DNA duplex, which is packed into chromatin, must be separated into two DNA single strands (ssDNA) before being replicated or transcribed, thus generating positive supercoils ahead the polymerases. DNA and RNA polymerases act in coordination with multiple enzymes and accessory factors, which include helicases that open the DNA duplex and topoisomerases that solve DNA topological constrains. Replication and transcription machineries are assembled at precise genomic locations, called origins, and promoters, respectively, and can travel for several DNA kilobases in 5′–3′ direction before being dismantled at termination sites. During transcription, several RNA polymerases transcribe one DNA strand, while the other remains transiently in single stranded conformation at the transcription bubble. During DNA synthesis, two replication machineries, called replisomes, move in opposite directions from one origin and duplicate both lagging and leading strands in a coordinated fashion, only once in each *S*-phase of the cell cycle. Replication and transcription compete for the same DNA template and can therefore interfere with each other. Transcription can arrest DNA synthesis and compromise replication fork stability, thus causing replication stress. Since the polarity of DNA and RNA synthesis is the same, a replication fork encounters the transcription machinery head-on on the lagging strand template and codirectionally on the leading strand. Although both types of collisions can disrupt or arrest replication forks *in vivo* (Deshpande and Newlon, 1996; Azvolinsky et al., 2009; Dutta et al., 2011; Merrikh et al., 2011; Alzu et al., 2012), several lines of evidence indicate that frontal clashes between replication and transcription mainly affect genome stability. The organization of bacterial genomes imparts in fact a co-orientation bias of replication and transcription of highly expressed and/or essential genes, thus avoiding deleterious head-on conflicts (Rocha, 2008). A preference for co-orientation of replication and transcription was also observed in human genome (Huvet et al., 2007). Moreover, head-on replication–transcription collisions are prevented by specific fork barriers at highly expressed ribosomal DNA (rDNA) in eukaryotic organisms (Kobayashi, 2014). From bacteria to humans, actively transcribed genes exhibit elevated spontaneous mutation and recombination rates, which are stimulated by replication (Prado and Aguilera, 2005; Kim et al., 2007; Gottipati et al., 2008; Paul et al., 2012). Transcription-associated recombination (TAR) and transcription-associated mutagenesis (TAM) increase when the lagging strand template is the transcribed strand (Prado and Aguilera, 2005; Kim et al., 2007; Paul et al., 2012), suggesting that head-on replication–transcription collisions are more detrimental to fork stability than codirectional ones. Protein–protein clashes on the lagging strand template, which contains ssDNA loop, would be particularly dangerous for fork integrity. Some evidence indeed suggests that the replisome may contact the transcription machinery (Mirkin and Mirkin, 2005). However, it is also possible that positive supercoils generated by polymerases moving toward each other, prevent a direct clash between them. In this case, fork arrest, and DNA damage may rather result from DNA topological constrains formation (Olavarrieta et al., 2002).

The replication fork has to face both nascent RNA and proteins, when it encounters the transcription machinery. RNA biogenesis proteins that co-transcriptionally process nascent RNA, including the splicing factor ASF/SF2, the THO/TREX mRNA export complex and the mRNA cleavage and polyadenylation machinery, prevent the re-hybridization of RNA to the transcribed DNA strand and therefore the formation of dangerous R-loop structures that could affect fork progression (Li and Manley, 2005; Gomez-Gonzalez et al., 2011; Wahba et al., 2011; Stirling et al., 2012). R-loop formation is favored by negative supercoiled DNA (Drolet, 2006), which accumulates behind the advancing RNA polymerase according to the twin-supercoiled domain model (Liu and Wang, 1987). It is therefore likely that the excess of positive supercoiled DNA accumulating in head-on encounters between the replisome and the transcription machinery may contribute to R-loop stabilization at the transcription bubble (Bermejo et al., 2012).

R-loops are physiological intermediates of several biological processes, including eukaryotic and prokaryotic immune responses or transcription termination (Skourti-Stathaki and Proudfoot, 2014). However, several studies from bacteria to humans suggest that uncontrolled accumulation of R-loops can affect genome integrity and proper chromatin organization, most likely by interfering with DNA synthesis.

## Mechanisms that Regulate Replication–Transcription Conflicts in Eukaryotic Cells

In prokaryotic cells, DNA synthesis starts at single origins of replication and since highly transcribed and/or essential genes are located on the leading strand template, harmful head-on conflicts between replication and transcription are prevented by genome organization (Rocha, 2008). Nevertheless, bacteria have evolved different strategies to resolve replication–transcription conflicts. These strategies relay on both auxiliary DNA helicases of the replisome that remove proteins and/or R-loops and transcription regulators that rescue stalled/backtracked RNA polymerases (Merrikh et al., 2012). Eukaryotic chromosomes are replicated from multiple origins differentially selected for firing, thus increasing the complexity of the replication–transcription interference.

**FIGURE 1:**
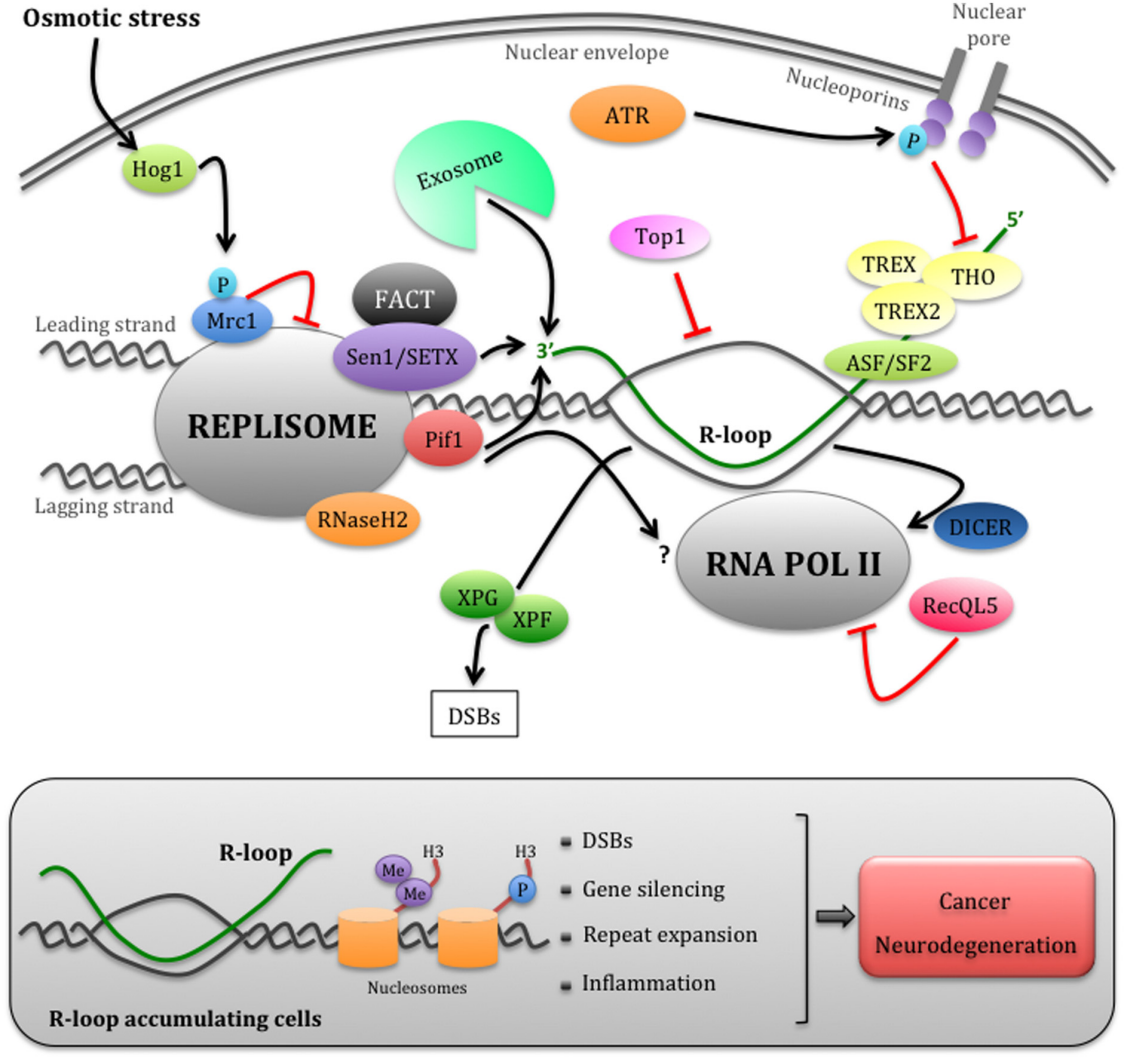
**Eukaryotic mechanisms that manage replication– transcription conflicts**. Schematic representation of a head-on encounter between the replisome and RNAPII. The cotrascriptional processing of nascent RNA, including its export through the nuclear envelope mediated by the THO/TREX and TREX2 complexes, can impede replication forks progression. The ATR checkpoint pathway temporarily inhibits RNA export by phosphorylating nucleoporins, thus allowing fork advancement. However, this process may generate harmful R-loop structures, more likely in head-on replication–transcription encounters. Multiple factors, including the accessory DNA/RNA helicases of the replisome Sen1/SETX and Pif1, the RNA exosome, RNaseH2, and Toposomerase I, may cooperate in limiting R-loop accumulation at the fork. The FACT complex, which interacts with SETX, could be involved in the re-establishment of chromatin status upon replication–transcription collisions. R-loops can be also processed into DSBs by XPG and XPF endonucleases. Hog1-dependent Mrc1 phosphorylation and RecQL5 modulate the speed of the replisome or RNAPII, respectively, while Dicer dislodges RNAPII at fork passage. Failure to promptly remove R-loops (gray box) causes not only DSBs, but also chromatin condensation through the accumulation of H3S10P and H3K9me2 markers, which contributes to fork arrest and gene silencing. Unrestrained R-loop accumulation has been also linked to repeats expansion and inflammation events, thus contributing to cancer and neurodegeneration (refer to text for further details).

Growing evidence suggests that the Ataxia telangiectasia mutated and Rad3-related (ATR) checkpoint kinase and downstream factors play a central role in coordinating replication with transcription. In mammals, the ATR pathway controls the stability of both common fragile sites (CFSs) and early replicating fragile sites (ERFSs), specific genomic regions prone to rearrangements under replication stress (Casper et al., 2002; Barlow et al., 2013). Some of these fragile elements correspond to R-loop accumulating long genes or highly transcribed genes (Helmrich et al., 2011; Barlow et al., 2013). Recent studies in budding yeast have suggested some mechanisms by which ATR pathway coordinates replication with transcription (**Figure 1**). The stress-activated protein kinase Hog1 phosphorylates Mrc1, a downstream component of the ATR pathway, and Mrc1 phosphorylation is crucial to slow down fork progression and to prevent TAR due to collisions with transcription (Duch et al., 2013). Moreover, the temporarily inhibition of transcription at fork passage mediated by the ATR pathway is a preferred mechanism to prevent replication–transcription collisions at both RNA Polymerase III (RNAPIII)- and RNA Polymerase II (RNAPII)-transcribed genes. ATR pathway actively controls the disassembly of the pre-initiation complex at tRNA genes (Nguyen et al., 2010) and assists fork progression and stability at RNAPII transcribed genes by inhibiting topological constrains caused by gene gating (Bermejo et al., 2011). This process, which is mediated by THO/TREX and TREX-2 complexes and nucleoporins, couples RNAPII transcription to mRNA export through the nuclear envelope (Blobel, 1985; Bermejo et al., 2012). Although required for gene expression, gene gating might aggravate transcription-associated topological problems, contributing to fork instability and it is therefore inhibited by ATR-dependent phosphorylation of nucleoporins (Bermejo et al., 2011). It has been proposed that, upon checkpoint-dependent gene gating inhibition, R-loop accumulation is favored at the twin supercoiled domain by head-on encounters between transcription and replication (Alzu et al., 2012; Bermejo et al., 2012). In this scenario, Topoisomerases I could be crucial to restrain R-loop accumulation by counteracting DNA negative supercoils (Tuduri et al., 2009).

Moreover, factors that remove transcription blocks and nascent RNA, similarly to what described for bacteria, could be also crucial to promote fork progression. Several helicases involved in replication fork stability maintenance, including HARP, WRN, BLM, Sen1, and Pif1, can remove RNA:DNA hybrids *in vitro* (Kim et al., 1999; Boule and Zakian, 2007; Popuri et al., 2008; Chakraborty and Grosse, 2011; Grierson et al., 2012; Kassavetis and Kadonaga, 2014). For yeast Pif1 and Sen1 DNA/RNA helicases a role in preventing replication–transcription interference has been also ascertained *in vivo*. In particular, members of the Pif1 helicase family assist fork progression through several type of natural barriers, including transcription blocks (Ivessa et al., 2003; Azvolinsky et al., 2009; Paeschke et al., 2011), while Sen1 is specifically required to prevent RNA:DNA hybrids accumulation at the fork in head-on encounters with RNAPII transcribed genes (Alzu et al., 2012). In human, both Senataxin/SETX, the ortholog of Sen1, and Aquarius, an helicase structurally related to Senataxin, prevent DNA damages caused by RNA:DNA hybrids accumulation, suggesting that this function has been evolutionarily conserved (Yuce and West, 2013; Sollier et al., 2014). Another suggested distinct role of Sen1/Senataxin is to promote transcription termination by removing R-loops (Mischo et al., 2011; Skourti-Stathaki et al., 2011). It is therefore possible that specific termination factors are engaged at fork passage to inhibit transcription. The above scenario is also consistent with the finding that the exosome, a multi-protein complex that degrades aberrant RNA molecules and is involved in transcription termination, co-localizes with Senataxin at R-loop-dependent nuclear foci in response to replication stress (Richard et al., 2013). The exosome could cooperate with Senataxin in removing R-loops by degrading RNA moiety (Richard et al., 2013).

RNaseH proteins, a class of enzymes that specifically degrades RNA in RNA:DNA hybrids, are also likely crucial to prevent replication–transcription interference. In budding yeast, RNaseH1 and RNaseH2 show specificity for R-loop resolution and RNaseH2 could have uniquely access to replication-associated RNA:DNA hybrids by interacting with the replisome (Wahba et al., 2011; Chon et al., 2013). Moreover, a yeast genome-wide study reported RNA:DNA hybrids accumulation at long or short genes in cells inactivated for RNaseH1/2 or Sen1, respectively (Chan et al., 2014). This data suggests that different anti-R-loop pathways could act at different genomic locations, even though how this is achieved remains unclear.

Finally, modulators of RNA polymerase activity have been involved in preventing replication–transcription collisions in eukaryotic cells. The human RecQL5 is a member of RecQ DNA helicases family that interacts with RNAPII (Aygun et al., 2008). RecQL5 acts as elongator factor to modulate RNAPII speed and to prevent chromosomal rearrangements across transcribed genes and certain CFSs (Saponaro et al., 2014). A recent study in fission yeast suggests another mechanism involving Dicer, a component of the RNA interference pathway (RNAi), in preventing transcription at putative sites of collisions with replication (Castel et al., 2014). Dicer, independently from the other components of the canonical RNAi pathway, promotes the release of RNAPII from the 3′ end of highly transcribed genes and from antisense transcribed rDNA regions. Transcription inhibition prevents the loss of rDNA repeats through homologous recombination. This specific function of Dicer at transcribed genes resembles the one of the RNAi pathway at pericentromeric regions, where it coordinates replication with transcription. In this way, RNAi pathway protects stalled forks from unscheduled homologous recombination, whose engagement interferes with the proper establishment of epigenetic modifications (Zaratiegui et al., 2011).

While it is evident that multiple pathways are involved in dealing with replication–transcription conflicts in eukaryotic cells (**Figure 1**), further studies are required to dissect their interconnections and the checkpoint-mediated regulation. These studies will be relevant to understand the causes of unrestrained R-loop accumulation that triggers both genetic and epigenetic instability in replication–transcription interference.

## Replication–Transcription Conflicts as a Cause of Genetic and Epigenetic Instability

Strong evidence from bacteria to humans indicates that transcription damage DNA by arresting replication fork progression. Indeed stalled replication forks accumulate ssDNA gaps and become prone to unscheduled recombination events and DNA double strand breaks (DSBs) formation. R-loops are thought to contribute to fork arrest, although the mechanism involved is unclear. An acknowledged model suggests that the RNA:DNA hybrid in the R-loop hampers fork progression, although R-loop bypass mechanisms could also be envisaged. Supporting this idea and consistent with *in vitro* observations in bacterial system, mRNA mediated re-priming of DNA synthesis at the leading strand has been observed during codirectional collisions with transcription (Pomerantz and O’Donnell, 2008). Moreover, another possible by-pass mechanism could relay on the uncoupling of leading and lagging strand synthesis and replication across the non-transcribed strand (Alzu et al., 2012). This pathway may require post-replication repair mechanisms, such as template switching and/or translesion DNA synthesis, for DNA replication across RNA:DNA hybrids (Gomez-Gonzalez et al., 2009). However, these by-pass processes may contribute to TAR and TAM occurrence, thus causing genome instability. The R-loop structure, which resembles the one of a D-loop recombination intermediate, could be processed by specific endonucleases leading to DSBs. In agreement with this idea, a recent study showed that in cells lacking Senataxin, Aquarius or the splicing factor ASF/SF2, unscheduled R-loops are processed into DSBs by XPG and XPF endonucleases, together with the Cockayne syndrome group B protein belonging to the transcription-coupled nucleotide excision repair (TC-NER) pathway (**Figure 1**; Sollier et al., 2014). These data clearly suggest that uncontrolled R-loop formation causes DSBs, although how R-loop processing by TC-NER pathway is coordinated with replication remains to be elucidated.

Recent findings suggested that pathological accumulation of R-loops and/or RNA:DNA hybrids is also linked to complex genomic rearrangements, including quasi-palindrome-associated mutations (Kim et al., 2013) and nucleotide repeat expansions, which underline several human neurological disorders (Lin et al., 2010; Groh et al., 2014; Haeusler et al., 2014; Loomis et al., 2014; Rigby et al., 2014).

In addition, uncoordinated replication–transcription clashes may also interfere with proper chromatin markers deposition, causing epigenetic instability (**Figure 1**). Chromatin markers are maintained through DNA replication by coupling the deposition of recycled parental histones to newly synthesized histones on duplicated DNA (Alabert and Groth, 2012). Defective fork progression at natural replication barriers such as G4 forming sequences leads to accumulation of repressive chromatin markers and gene silencing (Paeschke et al., 2011; Schiavone et al., 2014). Moreover, accumulation of stalled replication forks induced by treatment with the anticancer drug doxorubicin causes gene repression (Im et al., 2014). R-loops and/or RNA:DNA hybrids also influence the chromatin status. For instance, R-loop formation associates with unmethylated CpG island promoters in human genome (Ginno et al., 2012) and is linked to RNAi-directed heterochromatin formation at mammalian gene terminators (Skourti-Stathaki et al., 2014) and at centromeres in fission yeast (Nakama et al., 2012). While in these contexts R-loops promote a proper chromatin organization, it has been shown that an excessive R-loop accumulation at certain DNA regions induces unscheduled chromatin condensation. A recent study demonstrated that human, worm, or yeast cells depleted for Senataxin, RNaseH or the THO complex accumulate at transcribed genes both R-loops and phosphorylated H3 at S10 (H3S10P), a mitotic marker of chromatin condensation (Castellano-Pozo et al., 2013). R-loops are also linked to increased levels of the heterochromatin marker H3 dimetylated K9 (H3K9me2) in nematode cells inactivated for the THO complex (Castellano-Pozo et al., 2013). Furthermore, colocalization of R-loops and H3K9me2 has been also reported at the FXN gene expanded in Fragile X syndrome, likely contributing to its transcriptional silencing (Groh et al., 2014). It has been proposed that R-loop-mediated chromatin compaction would not only prevent transcription, but also contribute to impair fork progression (Castellano-Pozo et al., 2013). The idea that replication–transcription conflicts impact on chromatin structure is also supported by additional recent findings on the chromatin remodeling FACT complex, which physically interacts with Senataxin (Yuce and West, 2013; Hill et al., 2014). The FACT complex seems to be crucial to re-establish proper chromatin status after replication fork passage throughout transcribed DNA regions (Herrera-Moyano et al., 2014).

Altogether, it appears that failure to coordinate replication with transcription not only damages DNA, but also prevents gene expression, thus seriously affecting cell functionality.

## Replication–Transcription Conflicts: Implications for Cancer and Neurodegenerative Diseases

Replication stress is a hallmark of precancerous cells and is responsible for the gross chromosomal rearrangements observed in advanced tumors (Bartkova et al., 2006; Di Micco et al., 2006; Burrell et al., 2013). In human cells, transcription promotes oncogene-induced replication stress (Jones et al., 2012) and contributes to the expression of both ERFSs and CFSs, which match to regions of chromosomal abnormalities observed in cancer cells (Helmrich et al., 2011; Barlow et al., 2013). As mentioned above, recombinogenic RNA:DNA hybrids and/or R-loops are tightly connected to replication–transcription conflicts, suggesting that dysfunctions in R-loops metabolism contributes to cancer development (**Table 1**; Tuduri et al., 2010; Bermejo et al., 2012). This idea is supported by recent studies that have involved the well-characterized tumor suppressor genes BRCA1 and BRCA2 in R-loop processing (Bhatia et al., 2014; Hill et al., 2014; Hatchi et al., 2015). In particular, the finding that BRCA1 interacts with Senataxin and with factors crucial for fork recovery from replication stress, such as the FACT and the MMS22–TONSL complexes (Hill et al., 2014), raises the possibility that BRCA1 limits the replication–transcription conflicts.

**Table 1:** Eukaryotic factors that limit replication-transcription conflicts and/or R-loop accumulation.

Factors	Human diseases
***Kinases and checkpoint factors***
Hog1	
Mrc1	
ATR	Seckel syndrome (OMIM 210600)
***R-loop processing endonucleases***
XPG, XPF	Xeroderma pigmentosum (OMIM 278750)
***RNA degradation factors***
RNaseH1	
RNaseH2	Aicardi-Goutires syndrome (OMIM 610333, 610181, 610329)
Exosome	Pontocerebellar hypoplasia (OMIM 614678)
***RNA:DNA helicases***
Senataxin	ALS4 (OMIM 602433), AOA2 (OMIM 606002), cancer
BLM	Bloom syndrome (OMIM 210900)
WRN	Werner syndrome (OMIM 277700)
HARP	Schimke immunoosseous dysplasia (OMIM 242900)
PIF1	Breast cancer
Aquarius	
***RNA Polymerase II modulators***
Dicer	
RecQL5	Cancer
***R-loop associated chromatin remodeller***
FACT complex	Cancer
***mRNA biogenesis factors***
THO/TREX	Cancer
TREX-2	Cancer
ASF/SF2	Cancer
***Further anti R-loop factors***
BRCA1, BRCA2	Breast-ovarian cancer
Top1	Cancer

It is interesting to note that the most characterized factors that counteract R-loop accumulation have been implicated in neurological disorders (**Table 1**). Senataxin is mutated in juvenile forms of Ataxia and amyotrophic lateral sclerosis (ALS) (Chen et al., 2004; Moreira et al., 2004), while RNaseH2 in the neuroinflammatory Aicardi–Goutières disorder (Crow et al., 2006). The observation that replication and homologous recombination do not occur in neurons raises the question of whether replication-associated and recombinogenic R-loops contribute to neurodegeneration. It is possible that unscheduled accumulation of R-loops impacts on the functionality of glial cells, a population of cycling cells that interacts with neurons and whose dysfunctions contribute to neurodegeneration (Lobsiger and Cleveland, 2007). Furthermore, uncontrolled R-loop accumulation during neurogenesis could have long-term effects on genome integrity and gene expression in mature neurons. Indeed persistent RNA:DNA hybrids are associated to repeats instability, transcriptional silencing and replication-dependent DSBs accumulation at GAA/TTC tracts, which characterize Frederich Ataxia and Fragile X neurological syndromes (Zhang et al., 2012; Groh et al., 2014; Loomis et al., 2014). Stable RNA:DNA hybrids are also linked to GGGGCC-expansion in C9ORF72, the most common genetic alteration in the neurodegenerative disorders ALS and frontotemporal dementia (Reddy et al., 2010; Haeusler et al., 2014).

Of interest, recent findings suggested that RNA:DNA hybrids containing viral or bacterial derived sequences can stimulate the innate immune system response (Kailasan Vanaja et al., 2014; Mankan et al., 2014; Rigby et al., 2014). The identification of RNA:DNA hybrids as activators of innate immunity has obvious implications for autoimmune diseases, including Aicardi–Goutières neuroinflammatory disorder. Moreover, if RNA:DNA hybrids directly contribute to trigger chronic inflammations status, this would have broad implications for the onset of cancer and neurodegenerative diseases (**Figure 1**; Amor et al., 2014; Hagerling et al., 2014).

## Concluding Remarks

Almost 30 years of pioneering works in simple model organisms and recent studies in human cells have established that uncoordinated replication–transcription conflicts and unscheduled R-loop accumulation significantly contribute to cause genetic and epigenetic instability associated to replication stress, a pathological condition that alters chromosomal structure and functionality. In humans increasing evidence links the inactivation of factors that limit replication–transcription interference and R-loop formation with cancer and/or neurodegenerative disorders onset (**Table 1**). An accurate dissection of the molecular mechanisms that prevent transcription-induced replication stress could therefore provide a future framework for understanding the molecular basis of cancer and neurodegeneration.

## Conflict of Interest Statement

The Editor Alessandra Montecucco declares that, despite being affiliated to the same institution as the authors Alessandra Brambati, Arianna Colosio, Luca Zardoni, Lorenzo Galanti and Giordano Liberi, the review process was handled objectively and no conflict of interest exists. The authors declare that the research was conducted in the absence of any commercial or financial relationships that could be construed as a potential conflict of interest.
